# Arrangements of Resting State Electroencephalography as the Input to Convolutional Neural Network for Biometric Identification

**DOI:** 10.1155/2019/7895924

**Published:** 2019-06-02

**Authors:** Chi Qin Lai, Haidi Ibrahim, Mohd Zaid Abdullah, Jafri Malin Abdullah, Shahrel Azmin Suandi, Azlinda Azman

**Affiliations:** ^1^School of Electrical and Electronic Engineering, Engineering Campus, Universiti Sains Malaysia, 14300 Nibong Tebal, Penang, Malaysia; ^2^Department of Neurosciences, School of Medical Sciences, Universiti Sains Malaysia, 16150 Kubang Kerian, Kelantan, Malaysia; ^3^School of Social Sciences, Universiti Sains Malaysia, 11800 Pulau Pinang, Malaysia

## Abstract

Biometric is an important field that enables identification of an individual to access their sensitive information and asset. In recent years, electroencephalography- (EEG-) based biometrics have been popularly explored by researchers because EEG is able to distinct between two individuals. The literature reviews have shown that convolutional neural network (CNN) is one of the classification approaches that can avoid the complex stages of preprocessing, feature extraction, and feature selection. Therefore, CNN is suggested to be one of the efficient classifiers for biometric identification. Conventionally, input to CNN can be in image or matrix form. The objective of this paper is to explore the arrangement of EEG for CNN input to investigate the most suitable input arrangement of EEG towards the performance of EEG-based identification. EEG datasets that are used in this paper are resting state eyes open (REO) and resting state eyes close (REC) EEG. Six types of data arrangement are compared in this paper. They are matrix of amplitude versus time, matrix of energy versus time, matrix of amplitude versus time for rearranged channels, image of amplitude versus time, image of energy versus time, and image of amplitude versus time for rearranged channels. It was found that the matrix of amplitude versus time for each rearranged channels using the combination of REC and REO performed the best for biometric identification, achieving validation accuracy and test accuracy of 83.21% and 79.08%, respectively.

## 1. Introduction

Progress in information technology makes security a crucial aspect in protecting personal details and information. Therefore, authentication is needed to allow the correct individual to access this information [1]. Authentication can be divided into three categories: the knowledge-based, token-based, and biometric-based approaches [2]. Knowledge-based approach depends on information that has been set by users, such as personal identification number (PIN) and textual password. Token-based approach uses an object that a person owns, such as smart card and passport. Knowledge-based and token-based approaches have disadvantages in which the identifiers may be forgotten, misplaced, or stolen [3]. Pass card and password that are stolen can cause losses in financial and intellectual property. For example, perpetrators of fraud may attempt to obtain sensitive information such as passwords and credit card details for malicious reasons. In order to overcome these issues, a biometric-based approach has been introduced [4].

Biometrics-based approach depends on a person's identity [5]. The approach enables authentication based on physiological or behavioral features to recognize an individual, which cannot be replicated or stolen. Biometrics can be further divided into two categories, which are conventional biometrics [6] and cognitive biometrics [7]. Conventional biometrics use the physiological properties of the individual, including fingerprint, palm print, gait, and iris scan. Behavior characteristics such as voice and signature are also parts of conventional biometrics. In contrast with conventional biometrics, cognitive biometrics measures the human brain signals during emotional and cognitive brain conditions. The recorded signals are used as biometric traits.

Human brain plays an important role in controlling the actions and behavior of individuals. Every movement of our human body reflects the signals which are sent by the human brain [8]. In order to pick up signals from the human brain, different modalities are used to study the differences of brain signals to trigger various actions. Examples of brain signal representation that can be seen are functional magnetic resonance imaging (fMRI) and electroencephalography (EEG). Nevertheless, EEG has higher temporal resolution [9] and is able to directly measure brain activity. Therefore, EEG is a better option in cognitive biometrics as the procedures to obtain EEG are relatively practical and cheap. In addition, EEG is able to provide important information that can discriminate two individuals [10]. The analysis of EEG is more consistent as the signals recorded are more substantial and explicit [11]. Besides, EEG is universal, unique, and robust, making it suitable to be used as cognitive measures for biometric identification [12].

During the recording of EEG, certain tasks will be carried out by the subject in order to evoke responses from the human brain. The responses are picked up by the EEG. In recent years, resting state eyes closed (REC) and resting state eyes open (REO) are implemented in biometrics. Subjects closed their eyes when the EEG are recorded (i.e., REC), followed by eyes open (i.e., REO). Both REC and REO tasks are performed in resting state. REC and REO are commonly used as it is able to differentiate brain conditions of different individuals [13].

In the work by Choi et al. [14], they extracted features from the alpha activity of the EEG during REC and REO, as alpha power becomes stronger when eyes are closed [15, 16]. Their experimental results showed that the spatiospectral patterns of changed alpha activity are different for individuals and can perform identification efficiently. In the work of Thomas et al. [17], sample entropy features are extracted from delta, theta, alpha, beta, and gamma bands of the REC and REO EEG. It was shown that beta band entropy has the highest intersubject variability. In their work, power spectral density (PSD) is concatenated with the entropy features and improves the performance. In the work of Suppiah et al. [18], PSD was also extracted from REC and REO EEG as features, and a Fischer linear discriminant classifier was trained to perform identification. In addition, Lee et al. [19] also extracted spectral power, maximum power, and frequency of maximum power in the alpha band from the REC EEG in their design of the biometric authentication system. Fraschini et al. [20] proposed an REC REO EEG-based biometric system, which makes use of eigenvector centrality. Their work reported that the resting state functional brain network provides a better classification than only using a measure of functional connectivity. Their report strengthens the usability of resting state EEG as a biometric measure. It is observed from previous studies that resting state EEG provides important information which can differentiate individuals. In addition, resting state EEG can be applied to individuals who are severely ill as it only required their EEG acquisition in their resting state for biometric confirmation. From the review of many works, feature extraction is a complicated task as important information has to be selected to represent the individuals.

**Figure 1:**
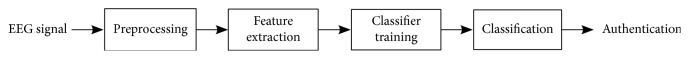
General framework of a biometric authentication system.

Based on the literature, we found that the general framework for biometric identification is as shown in Figure 1, which can be divided into four stages. The first stage is the preprocessing stage, where the raw signal will be preprocessed in order to remove unwanted elements such as noise and artifacts. The second stage is the feature extraction stage. In this stage, features are extracted from the preprocessed signal and then consequently used to train a classifier. If the size of the feature set is too huge, the feature dimension reduction will also be carried out at this stage. In the third stage, the feature set extracted from the second stage will be used to train a classifier. In the fourth stage, the trained classifier is used to perform classification for the input EEG.

There are several works that have been proposed for the EEG-based biometric authentication system using the framework in Figure 1. In the work of Koike-Akino et al. [21], blind source separation canonical correlation analysis (BSS-CCA) is used to preprocess the signal to remove ocular artifacts. Due to the huge dimensionality of their features, principal component analysis is used to rank and select the representative features from the feature pool. In their work, several machine learning classifiers were evaluated, and it was found that quadratic discriminant analysis (QDA) presents the best classification accuracy. He et al. [22] extract multivariate autoregressive (mAR) coefficients from multiple EEG channels as features. The extracted features are further hashed using the Fast Johnson-Lindenstrauss Transform- (FJLT-) based hashing algorithm to obtain compact hash vectors. The hash vectors are then used to train a Naive Bayes probabilistic model for identification.

Reshmi et al. [23] preprocessed the raw EEG signal using band pass filtering, baseline removal, detrending, and artifact removal. This is to remove unwanted components in the signal. Next, independent component analysis (ICA) is used to select the useful EEG components. The wavelet transform is used on the resultant signal to reveal the discriminative characteristic. The wavelet is used to train an artificial neural network (ANN) for identification. In the work by Thomas et al. [24], EEG signals are filtered using a zero-phase Butterworth filter and divided into five subfrequency bands. Entropy is computed from the resulting signals as features and used to train a Mahalanobis distance-based classifier.

The preprocessing is crucial to remove all the unwanted elements in a signal. However, it is time consuming to locate and remove the impurities in the signal. Impurities such as noises and artifacts can affect the training of classifiers and reduce the classification accuracy. In addition, determining the important features will also take up experimental time. In order to overcome the complex design of preprocessing, feature extraction and feature selection, CNN is one of the common methods used in development that requires classification [25].

CNN is a machine learning method which is inspired from the biological system [26]. The architecture is made up of multilayer perception (MLP) which consists of multiple hidden layers, combining the convolution layer and the conventional back propagation neural network dense layer. The hidden layers including the convolutional kernel in the CNN carry learnable parameters which require multiple iterations of learning and validation to determine the optimum value empirically [27]. The convolutional layers play the role of extracting important features from the input matrix through the weighted learnable kernels [28]. Each forward input of the matrix computes a feature map. The convolutional layers learn to activate the feature maps when the patterns of interest are detected in the input. Activated feature maps will be downsampled by using the pooling layer and further feed forward to the next layers. Fully connected layer (also known as dense layer) is trained using the feature map. The learning process of the learnable parameters implies backpropagation [29] and gradient decent [30].

**Figure 2:**
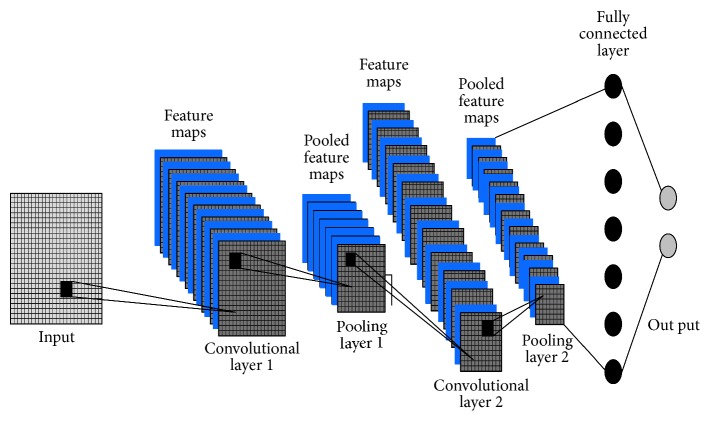
Example of the CNN structure.

An example of CNN structure is shown in Figure 2. The first convolutional layer will extract the features to form feature map from the output, followed by the first pooling layer. The first pooling layer will pool the features together and direct them into the second convolutional layer. The second convolutional layer extracts features to form the second feature map. Consequently, the second pooling layer will pool the features and direct it to the fully connected layer. From the fully connected layer, classification will be made and the input will be categorized into their labeled classes.

In recent years, CNN is frequently used in an EEG-based identification task [31–34]. In these approaches, input EEG is arranged in matrix form of amplitude versus time for every channel of EEG. The input matrix of amplitude versus time for every channel is a direct and convenient way to prepare the signals that can save input data preparation time. In the work by Ma et al. [34], an EEG-based biometric recognition was developed using CNN. In their work, resting state EEG is used as the input of CNN.

Resting state EEG lacks task-related features; hence, it is hard to perform feature extraction. CNN is one of the best used methods, which does not require preprocessing and feature extraction beforehand in order to obtain the feature set for the training of the classifier. In the work of Ma et al. [34], the CNN architecture used is shallow, which can avoid the possibility of overfitting. At the same time, their framework presents a high degree of accuracy for identification.

However, the input matrix of amplitude versus time might not be distinctive enough to represent the information in the signal to train the CNN. Besides the direct input of the raw EEG, there is no implementation of batch normalization in the work of Ma et al. [34] despite batch normalization is shown to be able to improve the performance of CNN [29].

The objective of this paper is to investigate the most suitable input to represent the EEG for the training of CNN. Six methods of organizing the input EEG are compared using the same data set and CNN architecture as in the work by Ma et al. [34]. The paper is divided into four sections.  Section 2 presents the methodology. Next, Section 3 presents the experimental results and discussion. Finally, the conclusion is given in Section 4.

## 2. Methodology

In this section, the methodology to investigate the most suitable input type of EEG and the effect of batch normalization are explained. In this work, six types of input of EEG are compared. The details of the input types will be explained in Section 2.3. In addition, the necessity of batch normalization is evaluated on all of the six input types. The input type of the work by Ma et al. [34] is used as the benchmark.

This section is divided into five subsections.  Section 2.1 will discuss the CNN architecture used in this study.  Section 2.2 presents the dataset used and the division of the dataset into training and testing sets. Section 2.3 explains the data preparation of EEG into six input types, and Section 2.4 is about batch normalization.

### 2.1. Convolutional Neural Network Architecture

For this biometric identification system, we have chosen the CNN architecture by Ma et al. [34] because their design of CNN is shallow, yet presents a high identification accuracy. In the work by Ma et al. [34], there is no implementation of batch normalization despite batch normalization was shown to be able to improve the performance of CNN [29]. Therefore, in this work, batch normalization will be included in the CNN architecture.

**Figure 3:**
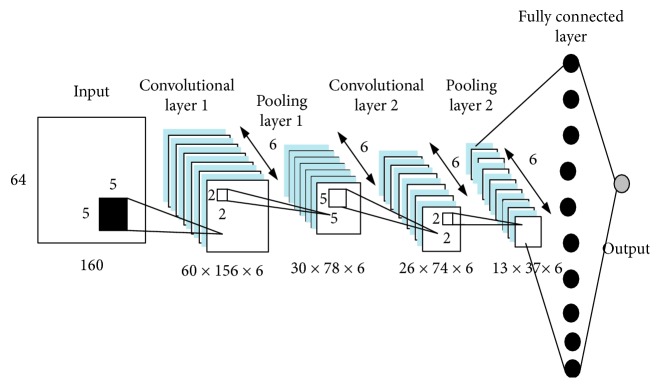
Structure of the CNN proposed by Ma et al. [34].

This CNN architecture is implemented to evaluate the performance of all six input types of EEG which will be further explained in Section 2.3. The CNN architecture is shown in Table 1 and Figure 3. The CNN is made up of five layers, including two convolutional layers, two pooling layers, and one fully connected layer. The input with size 64 × 160 will be directed to the first convolutional layer made up of six 5 × 5 filters, resulting an output of 60 × 156 × 6. The resulting feature map is then directed into an average pooling layer of size 2 × 2, producing an output of 30 × 78 × 6. The feature map is then directed to the second convolution layer made up of six 5 × 5 filters, outputting a feature map of 26 × 74 × 6. After going through an average pooling of size 2 × 2, an output of 13 × 37 × 6 is produced. This output is then flattened and directed into the fully connected layer. Softmax function is used as the activation function.

In this study, there are seven parameters that are fixed for a fair evaluation. These parameters are presented in Table 2. The learning rate of the CNN is set to 0.001 and remains constant throughout the training. In this study, the necessity of batch normalization is evaluated, and the result is shown in Section 3. *L*_2_ is used for batch normalization. The mini-batch size is set at 100 for experiments using REC or REO data set, while 200 are used for the combination of REC + REO. This is to allow a fair comparison of forming equal amount of epoch for training. The training repetition is fixed with 30 repetitions for all experiments. *L*_2_ regularization is used with the regularization factor of 0.0005. The optimizer used is the stochastic gradient decent with a momentum of 0.9.

### 2.2. Dataset

In this study, both REO and REC data used are from an open source dataset available on the website [35], which is the same dataset used in the work by Ma et al. [34]. The signals from the data set are recorded using 64-channel BCI2000 system with a sampling rate of 160 Hz. The dataset is divided into training set, testing set, and validation set. A total of 109 subjects' data are used under both REO and REC conditions. Recording time of the first 60 seconds is used for each condition. This is because the presence of more discriminating characteristics of the EEG is close to the beginning of the recording [36]. This signal is then divided into 60 subsets of one second each. After this division, 25 subsets will be used for training, five will be used for validation, and 30 will be used for testing. There will be three sample sets used, which are REO, REC, and REO combined with REC. There are a total of 2725 training samples, 545 validation samples, and 3270 testing samples for REC and REO, whereas for a combination of the REC and REO, it will have 5450 training samples, 1090 validation samples, and 6540 testing samples. In this study, subjects that are included in the dataset will be used for classification. Additional impostor subjects are not included.

### 2.3. Data Preparation

There are six types of input methods of the EEG compared in this study (Table 3). Three matrices and three images are compared. The three matrices are stored as images because images have manageable storage size.

#### 2.3.1. Matrix of Amplitude versus Time, *M*_1_

**Figure 4:**
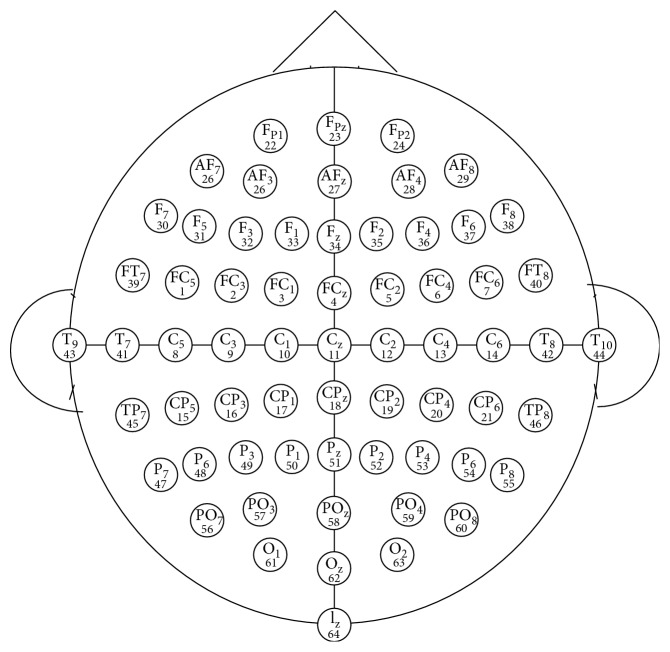
Default arrangement of EEG channels.

The first type of input is a matrix of amplitude versus time *M*_1_, as in the work of Ma et al. [34]. The arrangement of channels will use the default arrangement given by the dataset [35], as shown in Figure 4.

**Figure 5:**
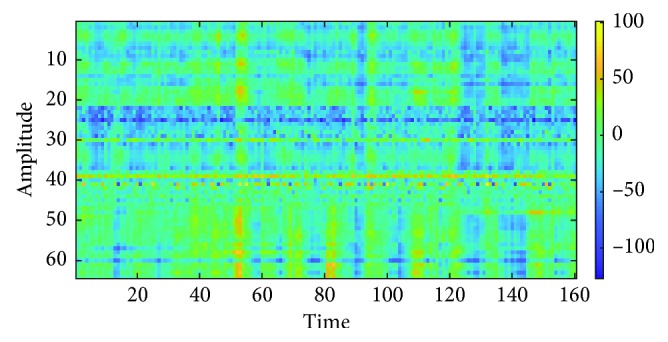
Matrix *M*_1_.

The matrix size is *N* × *F*_s_, where *N* is the number of channels and *F*_s_ is the sampling frequency. In this case, the matrix size will be 64 × 160 because the data are partitioned into one second segment, the number of channels is 64, and the sampling rate of EEG recording is 160 Hz. The components in the matrix are stored from the EEG by using the formula:

(1) $$\begin{array}{c} M_{1}\left(i,t\right)=x_{i}\left(t\right), \end{array}$$

where *i* is the channel of the sampling point, *t* is the time of the sampling point, and *x*_*i*_(*t*) is the amplitude of the sampling point of channel *i* at time *t*. The example for matrix *M*_1_ is shown in Figure 5.

#### 2.3.2. Matrix of Energy versus Time, *M*_2_

The second type of input data is the matrix of time versus energy *M*_2_. This input data matrix can be seen in the work of Sakhavil et al. [37] of motor imagery classification using CNN but is never used yet for EEG biometric identification. The Hilbert transform is applied on the input signal *x*(*t*) and computes the instantaneous energy for each of the time points in one second of EEG. Therefore, the matrix is 64 × 160. The Hilbert transform of the time series is calculated using the formula [38]:

(2) $$\begin{array}{c} H\left(x_{i}\left(t\right)\right)=\frac{1}{\pi }PV{\int^{}}_{\infty }^{-\infty }\frac{x_{i}\left(\tau \right)}{t-\tau }d\tau , \end{array}$$

where *x*_*i*_(*t*) is the input time series and PV is the Cauchy principal value. Next, the analytic EEG signal *z*_*i*_(*t*) is build from the Hilbert transform using the formula [38]:

(3) $$\begin{array}{c} z_{i}\left(t\right)=x_{i}\left(t\right)+jH\left(x_{i}\left(t\right)\right), \end{array}$$

where the real part of the signal *x*_*i*_(*t*) is the original value of the time point. The imaginary part *jH*(*x*_*i*_(*t*)) is the Hilbert transform of the time point.

The energy for channel *i* at a certain time point *t*, *E*_*i*,*t*_, is computed using the formula:

(4) $$\begin{array}{c} E_{i,t}={\left|H\left(x_{i}\left(t\right)\right)\right|}^{2}, \end{array}$$

where *H*(*x*_*i*_(*t*)) is the Hilbert transformed signal. The computed energy is stored in matrix *M*_2_ using the formula:

(5) $$\begin{array}{c} M_{2}\left(i,t\right)=E_{i,t}. \end{array}$$

**Figure 6:**
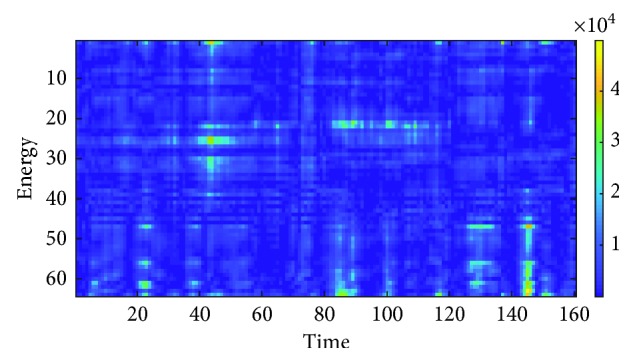
Matrix *M*_2_.

The example for matrix *M*_2_ is shown in Figure 6.

#### 2.3.3. Matrix of Amplitude versus Time for Rearranged Channels, *M*_3_

The third type of input data matrix is the rearrangement of channels according to the Pearson correlation coefficient, which was proposed by Wen et al. [39] for emotion recognition using CNN. Similar to *M*_2_, this data matrix has not been used in EEG biometric identification.

The Pearson correlation coefficient is the statistical value of linear correlation between two variables [40]. In their application, it is used to evaluate the relevant information between two electrodes (known as channel). The value of the coefficient ranges from −1 to 1, showing the negative linear correlation and positive linear correlation. Value 0 means there is no correlation between two variables. The Pearson correlation coefficient is computed for EEG using the formula:

(6) $$\begin{array}{c} \rho_{xy}=\frac{{\sum^{}}_{i=1}^{t}\left(x_{i}-\bar{x}\right)\left(y_{i}-\bar{y}\right)}{\sqrt{{\sum^{}}_{i=1}^{t}{\left(x_{i}-\bar{x}\right)}^{2}}\sqrt{{\sum^{}}_{i=1}^{t}{\left(y_{i}-\bar{y}\right)}^{2}}}, \end{array}$$

where *ρ*_*xy*_ is the computed coefficient, *x*_*i*_ and *y*_*i*_ are the two signals from different channels, $\bar{x}$ is the mean of signal *x*_*i*_, and $\bar{y}$ is the mean of signal *y*_*i*_.

**Figure 7:**
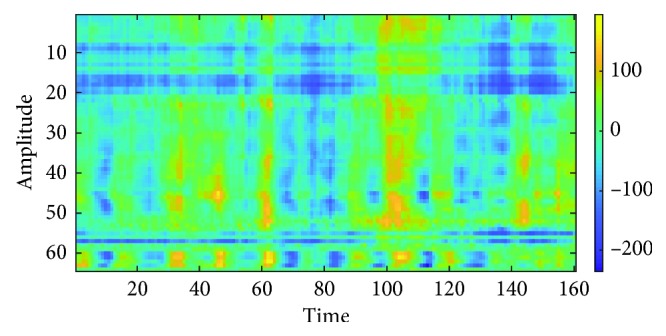
Matrix *M*_3_.

The rearrangement of channels started by placing channel one and the most correlated channel on the top most of the matrix. This step is then repeated for the rest of the channels, concatenating correlated channels next to each other. After the rearrangement, the amplitude of each sampling points is stored in matrix *M*_3_ using equation (1). The matrix size will be 64 × 160. The example for matrix *M*_3_ is shown in Figure 7.

#### 2.3.4. Image of Amplitude versus Time, *I*_1_

**Figure 8:**
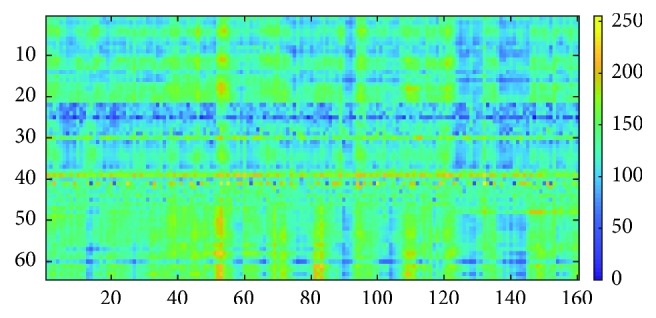
Image *I*_1_.

After the computation of matrix *M*_1_, each value of its components is scaled from 0 to 255 and stored as image *I*_1_. Storing EEG in image format makes it more feasible as it requires smaller storage space. However, there are no existing work in EEG-based identification that stores EEG in image format. The matrix is stored as an image using the formula:

(7) $$\begin{array}{c} I_{1}\left(i,t\right)=\frac{M_{1}\left(i,t\right)-M_{1_{\text{min}}}}{M_{1_{\text{max}}}-M_{1_{\text{min}}}}\times 255, \end{array}$$

where *i* is the channel of the sampling point, *t* is the time of the sampling point, *M*_1_max__ is the maximum value in *M*_1_, and *M*_1_min__ is the minimum value in *M*_1_. The example for image *I*_1_ is shown in Figure 8.

#### 2.3.5. Image of Energy versus Time, *I*_2_

**Figure 9:**
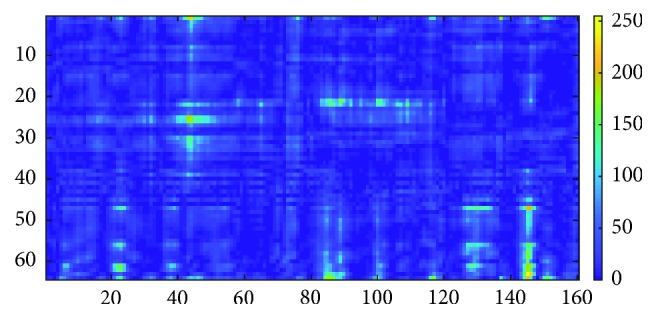
Image *I*_2_.

To form an image of matrix *M*_2_, the values in the matrix are scaled from 0 to 255 and stored in image *I*_2_ using the formula:

(8) $$\begin{array}{c} I_{2}\left(i,t\right)=\frac{M_{2}\left(i,t\right)-M_{2_{\text{min}}}}{M_{2_{\text{max}}}-M_{2_{\text{min}}}}\times 255, \end{array}$$

where *i* is the channel of the sampling point, *t* is the time of the sampling point, *M*_2_max__ is the maximum value in *M*_2_, and *M*_2_min__ is the minimum value in *M*_2_. The example for image *I*_2_ is shown in Figure 9. Similarly to image *I*_2_, there are no existing works in EEG-based identification that computes energy from EEG and stored in image format. It is suggested that the image format is easier for data storage and management.

#### 2.3.6. Image of Amplitude versus Time for Rearranged Channels, *I*_3_

**Figure 10:**
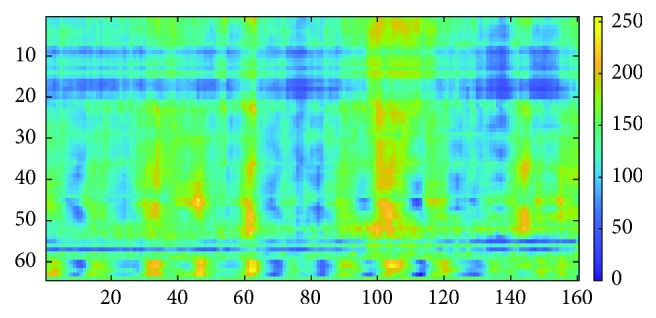
Image *I*_3_.

By referring to matrix *M*_3_, image *I*_3_ is formed. In the literature, there are no approaches in EEG-based identification that stored EEG matrices in image format. It is predictable that image of the EEG can ease researchers to manage the storage in a convenient way. The values in the matrix are scaled from 0 to 255 and stored in image *I*_3_ using the formula:

(9) $$\begin{array}{c} I_{3}\left(i,t\right)=\frac{M_{3}\left(i,t\right)-M_{3_{\text{min}}}}{M_{3_{\text{max}}}-M_{3_{\text{min}}}}\times 255, \end{array}$$

where *i* is the channel of the sampling point, *t* is the time of the sampling point, *M*_3_max__ is the maximum value in *M*_3_, and *M*_3_min__ is the minimum value in *M*_3_. The example for image *I*_3_ is shown in Figure 10.

### 2.4. Batch Normalization

In the work of LeCun et al. [29], batch normalization is introduced for network regularization [41]. In their work, batch normalization is applied to avoid overfitting of the network and to have better conservation of information throughout the training process.

Firstly, mean *μ*_*β*_ and variance *σ*_*i*_^2^ are calculated for the inputs mini-batch *x*_*i*_ to the batch normalization layer. Next, the mean and variance are used to calculate the normalized activations $\hat{x_{i}}$ using the formula:

(10) $$\begin{array}{c} {\hat{x}}_{i}=\frac{x_{i}-\mu_{\beta }}{\sqrt{\sigma_{i}^{2}+\epsilon }}, \end{array}$$

where *ϵ* is a constant that improves numerical stability when the variance of the mini-batch is small. In order to allow inputs with zero mean and unit variance that are not optimal for the layer, the batch normalization layer shifts and scales the activations. The scaled activation *z*_*i*_ is calculated using the formula:

(11) $$\begin{array}{c} z_{i}=\gamma {\hat{x}}_{i}+\beta , \end{array}$$

where *β* is the offset and *γ* is the scale factor. Both the parameters are learnable during training of the CNN. When the training is done, mean and variance are calculated for the full training set and then stored. While predicting using the trained CNN, the trained mean and variance will be used to normalize the activation.

Experiments using six types of input data are repeated by adding batch normalization layers in between the convolution layer and pooling layer.

## 3. Results and Discussion

The performance of each type of input is measured using two identification accuracies in terms of percentage, which are the validation accuracy and testing accuracy. The identification accuracies are obtained using a threefold cross validation. Both of the identification accuracies are calculated using the formula:

(12) $$\begin{array}{c} \text{accuracy}=\frac{\sum^{}C_{\text{P}}}{\sum^{}N_{\text{P}}}, \end{array}$$

where *C*_P_ is the correct prediction and *N*_P_ is the number of prediction done. Each of the identification accuracies is tabulated.

First, Table 4 shows the identification accuracy using the REC dataset. Among the input types, matrix *M*_1_ showed the highest validation and test accuracies, which are 74.86% and 67.04%, respectively. The image *I*_1_ presents validation and test accuracies which are slightly below matrix *M*_1_. This scenario shows that there was some information lost in between the conversion of matrix to an image. On the other hand, converting *M*_2_ into image *I*_2_ improved the validation and test accuracies. The values of power computed are large, costing complex calculation in the training of CNN. The conversion of matrix to image scaled the values into the range from 0 to 255, which normalized the larger values in the matrix.

At the same time, it was found that rearrangement of the channels according to the correlation is ineffective in representing the EEG. The implementation of image and matrix both shows validation and test accuracies lower than 40%. It is worth noting that channel reorganization alters the 2D spatial information presented by the matrix or the image. As our convolutional layers in CNN are using 2D filters, different features are extracted by these layers from the rearrangement of the channels, as compared to those extracted from the original EEG sequence. During the rearrangement of channels, important edges and patterns represented by the original EEG are lost, and there are no discriminating features that can be extracted during the training of CNN, thus causing low validation and testing accuracy.

The identification accuracy using the REO dataset is tabulated in Table 5. From Table 5, matrix *M*_1_ presented the highest validation and test accuracy. Mapping the original data from EEG into a matrix preserves the information for each individual. Similarly, image *I*_2_ has higher validation and test accuracy than matrix *M*_2_ due to the normalized values in the matrix-to-image conversion. Rearranging the channels of REO EEG shows similar validation and test accuracy with the REC, which is relatively low. Both *M*_3_ and *I*_3_ show validation and test accuracy lower than 30%.

Table 6 presents the identification accuracy, using the dataset of REO combined with REC. In using the REO + REC dataset, matrix *M*_1_ also presents the highest identification accuracy. It shows that storing raw EEG in matrix format effectively brings out the important information for the training of CNN. For the computation of power for the raw EEG, image *I*_2_ outperformed matrix *M*_2_ due to the normalization of the values. From the result, the implementation of rearranging the EEG channels is not effective, even with using the combination of REO and REC.

To have a better analysis of the best input type for each dataset, their identification accuracies are tabulated in Table 7. It can be seen that matrix *M*_1_ shows the best performance among each dataset used. The raw EEG contains patterns and information which can discriminate each subject. Such a property makes EEG a suitable trait for biometrics. In comparison to using REC or REO dataset itself, the combination of both achieved a higher validation and test accuracy. The combination of the REC and REO provides extra information for the training of CNN, making the trained model to be more robust towards different individuals.

By converting matrix *M*_1_ into an image, it can ease the dataset management by implementing into other existing structures of machine learning which are image-based implementation. From the results, image *I*_1_ did not perform as well as matrix *M*_1_. However, in the use of the REO + REC dataset, image *I*_1_ presented an elevated validation and test accuracy of 78.66% and 72.31%, respectively. It is suggested that implementing image *I*_1_ using the REO + REC dataset for biometrics is acceptable for easier handling of large dimension datasets.

## 4. Conclusion

From the experiment, arranging raw EEG in the form of matrix presents the best identification accuracy by using the REO + REC dataset. Referring to this, it is noticeable that resting states eyes close and eyes open should be used together as a trait for biometrics. Conversion of matrix to image can cause information loss, but when using the REO + REC dataset, it presented an acceptable range of results. Processing image form of raw EEG provides advantages such as less storage space, easier storage management, and dataset sharing among researchers. In addition, it was found that by rearranging the channels according to their correlation removes patterns and information from the raw EEG, which is not suitable for the application of EEG biometrics. It was also found that scaling and storing the power computed from the raw EEG into image form can improve the identification accuracy. This is because huge power values are normalized when they are rescaled into a range from 0 to 255 in the conversion process. This study concludes that matrix form of raw REO + REC EEG is sufficient for a robust biometric identification system.

## Figures and Tables

**Table 1:** Layers of the CNN and kernel size proposed by Ma et al. [34].

Index	Layer	Kernel size
1	Convolution layer	5 × 5
2	Average pooling layer	2 × 2
3	Convolution layer	5 × 5
4	Average pooling layer	2 × 2
5	Fully connected layer	—

**Table 2:** Parameters and values.

Parameter	Value
Learning rate	0.001
Batch normalization	*L*_2_ normalization
*L*_2_ Regularization	0.0005
Mini-batch size	100 (REO or REO), 200(REC + REO)
Optimizer	Stochastic gradient decent
Training repetitions	30
Momentum	0.9

**Table 3:** Type of input methods.

Input	Type	Size	Description
*M*_1_	Matrix	64 × 160	Matrix of amplitude versus time
*M*_2_	Matrix	64 × 160	Matrix of energy versus time
*M*_3_	Matrix	64 × 160	Matrix of amplitude versus time for rearranged channels
*I*_1_	Image	64 × 160	Image of amplitude versus time
*I*_1_	Image	64 × 160	Image of energy versus time
*I*_1_	Image	64 × 160	Image of amplitude versus time for rearranged channels

**Table 4:** Identification accuracy using the REC dataset.

Type of input REC	Validation accuracy (%)	Test accuracy (%)
*M*_1_	74.86	67.04
*M*_2_	37.80	37.21
*M*_3_	30.40	25.45
*I*_1_	62.64	59.80
*I*_2_	59.39	52.56
*I*_3_	19.88	16.01

**Table 5:** Identification accuracy using the RE0 dataset.

Type of input REC	Validation accuracy (%)	Test accuracy (%)
*M*_1_	81.47	74.10
*M*_2_	44.22	38.87
*M*_3_	29.88	25.80
*I*_1_	65.81	59.03
*I*_2_	64.95	56.67
*I*_3_	19.33	16.16

**Table 6:** Identification accuracy using the RE0 + REC dataset.

Type of input REC	Validation accuracy (%)	Test accuracy (%)
*M*_1_	83.21	79.08
*M*_2_	46.24	43.83
*M*_3_	31.10	29.27
*I*_1_	78.66	72.31
*I*_2_	67.19	60.82
*I*_3_	18.48	15.85

**Table 7:** Identification accuracy of best performance input types for each dataset.

Type of input	Dataset	Validation accuracy (%)	Test accuracy (%)
*M*_1_	REC	74.86	67.04
*M*_1_	REO	81.47	74.10
*M*_1_	REO + REC	83.21	79.08

## Data Availability

Previously reported EEG data were used to support this study and are available at https://www.physionet.org/physiobank/database/eegmmidb. These prior studies (and datasets) are cited at relevant places within the text as references [35]. The data used to support the findings of this study are available from the corresponding author upon request.
